# Biomolecular Predictors of Recurrence Patterns and Survival in IDH-Wild-Type Glioblastoma: A Retrospective Analysis of Patients Treated with Radiotherapy and Temozolomide

**DOI:** 10.3390/brainsci15070713

**Published:** 2025-07-02

**Authors:** Paolo Tini, Flavio Donnini, Francesco Marampon, Marta Vannini, Tommaso Carfagno, Pierpaolo Pastina, Giovanni Rubino, Salvatore Chibbaro, Alfonso Cerase, Giulio Bagnacci, Armando Perrella, Maria Antonietta Mazzei, Alessandra Pascucci, Vincenzo D’Alonzo, Anna Maria Di Giacomo, Giuseppe Minniti

**Affiliations:** 1 Unit of Radiation Oncology, Department of Medicine, Surgery and Neurosciences, University of Siena, 53100 Siena, Italy; flavio.donnini@student.unisi.it (F.D.); m.vannini@ao-siena.toscana.it (M.V.);; 2 Radiation Oncology, Policlinico Umberto I, Department of Radiological, Oncological and Pathological Sciences, “Sapienza” University of Rome, 00185 Rome, Italygiuseppe.minniti@unisi.it (G.M.); 3 Neurosurgery, Azienda Ospedaliera Universitaria Senese, 53100 Siena, Italy; salvatore.chibbaro@unisi.it; 4 Unit of Neuroradiology, Azienda Ospedaliera Universitaria Senese, 53100 Siena, Italy; alfonso.cerase@ao-siena.toscana.it; 5 Unit of Diagnostic Imaging, Department of Medicine, Surgery and Neurosciences, University of Siena, 53100 Siena, Italy; giulio.bagnacci@unisi.it (G.B.); armando.perrella@unisi.it (A.P.); mariaantonietta.mazzei@unisi.it (M.A.M.); 6 Center for Immuno-Oncology, Department of Medicine, Surgery and Neurosciences, University of Siena, 53100 Siena, Italy; ale.pascucci@ao-siena.toscana.it (A.P.); dalonzovincenzo94@gmail.com (V.D.); annamaria.digiacomo@unisi.it (A.M.D.G.); 7 IRCSS Neuromed, 86077 Pozzilli, Italy

**Keywords:** glioblastoma, radiotherapy, pattern of recurrence, EGFR, MGMT

## Abstract

Background and Aim: Glioblastoma (GBM) is the most aggressive primary brain tumor in adults, with poor prognosis despite maximal surgical resection, radiotherapy (RT), and temozolomide (TMZ) per the Stupp protocol. IDH-wild-type GBM, the predominant molecular subtype, frequently harbors EGFR amplification and is resistant to therapy, while MGMT promoter methylation predicts improved TMZ response. This study aimed to assess the prognostic impact of EGFR and MGMT status on survival and recurrence patterns in IDH-wild-type GBM. Materials and Methods: We retrospectively analyzed 218 patients with IDH-wild-type GBM treated at the Azienda Ospedaliero-Universitaria Senese (2016–2024). All patients underwent maximal safe surgical resection whenever feasible. The cohort includes patients who received gross total resection (GTR), subtotal resection (STR), or biopsy only, depending on tumor location and clinical condition, followed by intensity-modulated RT (59.4–60 Gy) with concurrent and adjuvant TMZ. EGFR amplification was assessed via FISH/NGS and immunohistochemistry; MGMT promoter methylation was determined using methylation-specific PCR. Progression-free survival (PFS), overall survival (OS), and recurrence patterns (in-field, marginal, out-field) were evaluated using Kaplan–Meier, Cox regression, and logistic regression analyses. Results: Among patients (64.7% male; mean age 61.8), 58.7% had EGFR amplification and 49.1% showed MGMT methylation. Median OS and PFS were 14 and 8 months, respectively. EGFR non-amplified/MGMT methylated tumors had the best outcomes (OS: 22.0 months, PFS: 10.5 months), while EGFR-amplified/MGMT unmethylated tumors fared worst (OS: 10.0 months, PFS: 5.0 months; *p* < 0.001). MGMT methylation was an independent positive prognostic factor (HR: 0.48, *p* < 0.001), while EGFR amplification predicted worse survival (HR: 1.57, *p* = 0.02) and higher marginal recurrence (OR: 2.42, *p* = 0.01). Conclusions: EGFR amplification and MGMT methylation significantly influence survival and recurrence dynamics in IDH-wild-type GBM. Incorporating these biomarkers into treatment planning may enable tailored therapeutic strategies, potentially improving outcomes in this challenging disease. Prospective studies are needed to validate biomolecularly guided management approaches.

## 1. Introduction

Glioblastoma (GBM) is the most common and aggressive primary malignant brain tumor in adults, accounting for approximately 50% of all primary central nervous system malignancies [1,2]. The annual incidence is estimated at 3.27 cases per 100,000 individuals, with a median age at diagnosis of 66 years and a higher prevalence in males [3]. Despite advancements in surgical and adjuvant treatments, including radiotherapy (RT) and temozolomide (TMZ) as per the Stupp protocol which consists of concurrent RT and daily TMZ followed by adjuvant TMZ for six cycles [4,5], prognosis remains poor, with a median overall survival (OS) of approximately 15 months and a five-year survival rate of less than 5% [6,7].

The 2021 WHO classification emphasizes the molecular heterogeneity of glioblastomas, particularly the distinction between IDH-mutant and IDH-wild-type tumors [8]. IDH-wild-type GBM, which lacks mutations in IDH1/IDH2, is the predominant subtype and exhibits a highly infiltrative and therapy-resistant phenotype [9,10]. Key molecular alterations include EGFR amplification, TERT promoter mutations, and chromosome 7 gain/chromosome 10 loss, all of which contribute to tumor progression and treatment resistance [11,12].

These molecular characteristics have important implications for prognosis and therapeutic response. Biomolecular markers have become integral to risk stratification in GBM [13]. EGFR alterations, including amplification and the EGFRvIII mutation, are associated with aggressive tumor biology and resistance to therapy [14,15]. MGMT promoter methylation, an epigenetic modification that silences a key DNA repair enzyme, is a well-established predictor of improved response to TMZ, prolonging OS and progression-free survival (PFS) [5,16].

Despite these insights, gaps remain in understanding how these biomarkers influence recurrence patterns in GBM [17]. Tumor recurrence typically occurs within or near the initial treatment volume, however, the biological determinants of in-field, marginal, or out-field recurrence remain poorly defined [18]. Identifying correlations between molecular alterations and recurrence patterns could improve radiotherapy target delineation and optimize personalized treatment strategies [19,20].

This study aims to investigate the prognostic significance of EGFR, MGMT, in patients with IDH-wild-type GBM treated with RT and TMZ according to the Stupp protocol. Specifically, we will evaluate the impact of these biomarkers on OS, PFS, and recurrence patterns. By integrating biomolecular profiling with clinical outcomes, this research may contribute to more tailored treatment approaches and improved therapeutic decision-making for patients with this highly aggressive malignancy.

## 2. Study Design and Patient Selection

This retrospective cohort study included patients with histologically confirmed IDH-wild-type glioblastoma (GBM) who received radiotherapy (RT) and temozolomide (TMZ) treatment at the Radiotherapy Department of the Azienda Ospedaliero-Universitaria Senese between January 2016 and February 2024. Patients were eligible for inclusion if they had a confirmed diagnosis of IDH-wild-type GBM, completed standard chemo-radiotherapy according to the Stupp protocol, and had complete clinical, radiological, and biomolecular data. Patients enrolled in experimental clinical trials or those with incomplete follow-up data were excluded.

### 2.1. Data Collection and Treatment Approach

Clinical, biomolecular, and radiotherapeutic data were extracted from institutional records. Clinical parameters included age, sex, and performance status, assessed using the Karnofsky Performance Score (KPS) at diagnosis. The extent of surgical resection was categorized as gross total resection (GTR), subtotal resection (STR), or biopsy.

Biomolecular analysis was performed on tumor samples obtained during surgery. EGFR status was evaluated both at the genomic and protein expression level. Gene amplification was assessed using fluorescence in situ hybridization (FISH) or next-generation sequencing (NGS), while protein expression was analyzed through immunohistochemistry (IHC) using monoclonal anti-EGFR antibodies. Patients were classified into two groups:

1. EGFR-amplified tumors with EGFR protein overexpression.
2. Non-amplified (wild-type) tumors with normal, weak, or absent EGFR expression.

Additionally, MGMT promoter methylation was determined using methylation-specific PCR (MS-PCR), with tumors showing >5% methylation classified as methylated.

All patients underwent maximal safe surgical resection whenever feasible. The cohort includes patients who received gross total resection (GTR), subtotal resection (STR), or biopsy only, depending on tumor location and clinical condition. Radiotherapy was delivered using intensity-modulated radiotherapy (IMRT), with a total dose of 59.4–60 Gy, administered in 1.8–2 Gy fractions over six to seven weeks. Concurrent TMZ was prescribed at 75 mg/m^2^/day during RT, followed by adjuvant TMZ at 150–200 mg/m^2^/day for five days every 28-day cycle [4,7]. Treatment volumes were defined based on imaging. The gross tumor volume (GTV) included the surgical cavity and residual contrast-enhancing tumor on T1-weighted MRI. The clinical target volume (CTV) was delineated with a 1.5 cm margin around the GTV, adjusted for anatomical barriers. The planning target volume (PTV) extended the CTV by an additional 3 mm to account for patient movement and setup variability.

### 2.2. Outcome Measures and Recurrence Classification

Patients underwent regular clinical and radiological follow-ups, with MRI scans performed every three months or sooner if progression was clinically suspected. Progression-free survival (PFS) was defined as the time from RT initiation to tumor progression or death, while overall survival (OS) was calculated from RT initiation to death from any cause.

**Figure 1:**
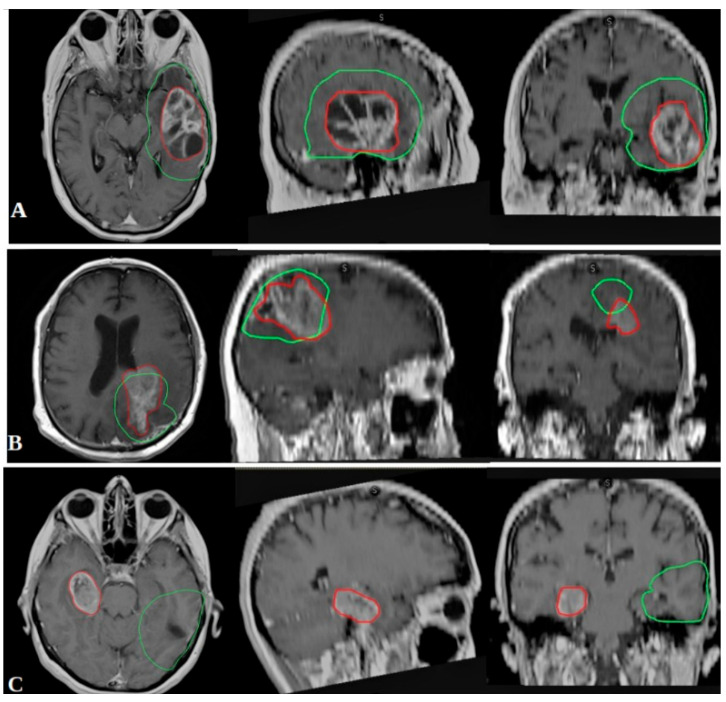
Pattern of relapse classification. Recurrence patterns were evaluated by co-registering follow-up MRI scans with RT treatment plans using RayStation^®^ software (RaySearch Laboratories AB, Stockholm, Sweden) and were categorized based on spatial distribution in relation to the 95% isodose line. Spatial recurrence: (**A**) in-field; (**B**) marginal; (**C**) Out-field. Green: PTV; Red: RELAPSE.

Recurrence patterns were evaluated by co-registering follow-up MRI scans with RT treatment plans using RayStation^®^ software version 2024B (RaySearch Laboratories AB, Stockholm, Sweden). Tumor progression was classified according to the Response Assessment in Neuro-Oncology (RANO) criteria [21], distinguishing complete response, partial response, stable disease, and progressive disease. Progression was confirmed through subsequent MRI at least two months after initial detection. Recurrences were categorized based on spatial distribution in relation to the 95% isodose line (Figure 1):

- In-field recurrence: ≥80% of the recurrent tumor volume within the 95% isodose line.
- Marginal recurrence: 20–80% of the recurrent volume within the 95% isodose line.
- Out-field recurrence: <20% of the recurrent volume within the 95% isodose line.

### 2.3. Statistical Analysis

Survival analysis was performed using the Kaplan–Meier method to estimate OS and PFS, while multivariate Cox regression analysis was applied to identify independent prognostic factors. Chi-square tests (χ^2^) were used to compare categorical variables, and Pearson’s correlation was applied to assess relationships between continuous variables. Logistic regression was conducted to explore associations between biomolecular markers and recurrence patterns. A *p*-value <0.05 was considered statistically significant. All statistical analyses were performed using IBM^®^ SPSS^®^ Statistics (version 21).

### 2.4. Ethical Considerations

Ethical approval was granted by the local Ethics Committee under protocol GLIOMARKERSOBS_2025-1, approved on 6 May 2025. All patients provided informed consent, and data were anonymized to ensure confidentiality in accordance with ethical guidelines.

## 3. Results

### 3.1. Patient Demographics and Clinical Characteristics

Of 312 patients initially evaluated between 2016 and 2024, 218 patients with histologically confirmed IDH-wild-type glioblastoma (GBM) met inclusion criteria after excluding those with incomplete clinical, radiological, or biomolecular data. The cohort comprised 141 males (64.7%) and 77 females (35.3%), with a mean age of 61.8 years (SD: 11.16). The majority of patients (88.5%) had a Karnofsky Performance Status (KPS) of 80–100, while 11.5% had a KPS below 80. Regarding surgical management, 28.4% underwent gross total resection (GTR), whereas 71.6% underwent subtotal resection (STR) or biopsy (Table 1).

### 3.2. Biomolecular Markers and Grouping Strategy

Patients were classified into four distinct groups based on EGFR status (amplified vs. non-amplified) and MGMT promoter methylation status (methylated vs. unmethylated):

1. EGFR non-amplified/MGMT methylated (EGFR neg/MGMT met);
2. EGFR non-amplified/MGMT unmethylated (EGFR neg/MGMT unm);
3. EGFR amplified/MGMT methylated (EGFR iper/MGMT met);
4. EGFR amplified/MGMT unmethylated (EGFR iper/MGMT unm).

Among the study population, high EGFR expression was observed in 58.7% of cases, while MGMT promoter methylation was detected in 49.1%.

### 3.3. Radiological Response and Disease Progression

Response to treatment was assessed to up 6 months after therapy completion (Table 2). The EGFR neg/MGMT met group exhibited the highest complete response (CR) rate (43.9%), whereas the EGFR iper/MGMT unm group had the lowest (11.8%). Partial response (PR) was recorded in 31.7% of EGFR neg/MGMT met patients, but only 9.4% of EGFR neg/MGMT unm patients. The progressive disease (PD) rate was highest in EGFR neg/MGMT unm (50.0%) and EGFR iper/MGMT unm (50.0%), compared to 11.8% in EGFR neg/MGMT met patients.

**Figure 2:**
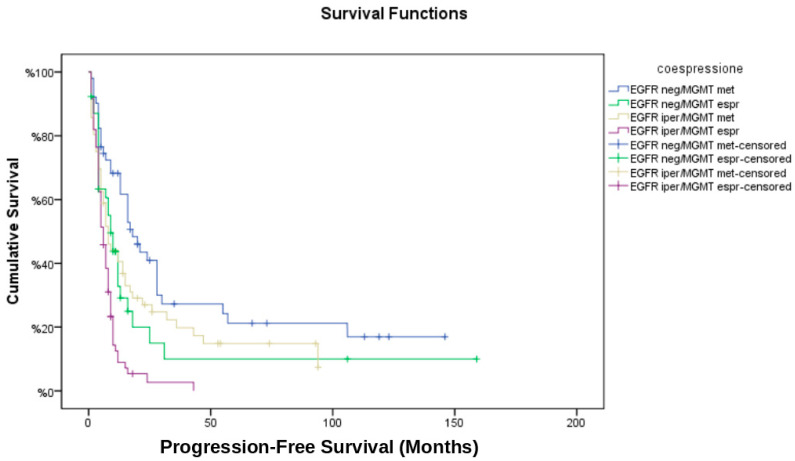
Kaplan–Meier curves for progression-free survival (PFS) across EGFR/MGMT molecular subgroups. Progression-free survival curves stratified by combined EGFR amplification (amplified vs. non-amplified) and MGMT promoter methylation (methylated vs. unmethylated) status. log-rank test: *p* < 0.001.

**Figure 3:**
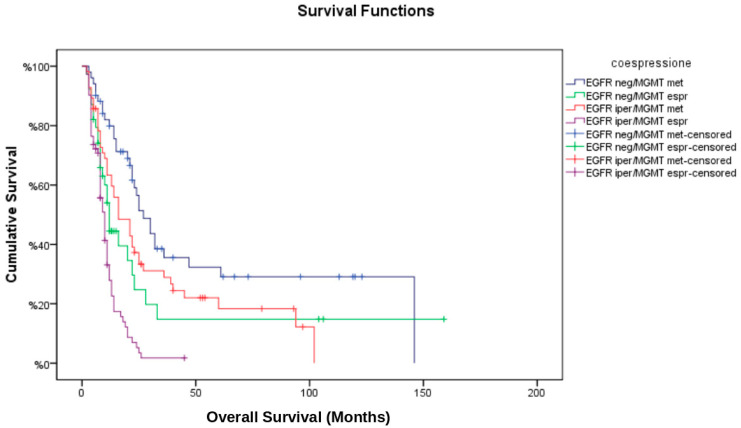
Kaplan–Meier curves for overall survival (OS) across EGFR/MGMT subgroups. Overall survival curves stratified by combined EGFR amplification (amplified vs. non-amplified) and MGMT promoter methylation (methylated vs. unmethylated) status. Log-rank test: *p* < 0.001.

The median progression-free survival (PFS) for the entire cohort was 8 months, while median overall survival (OS) was 14 months. Kaplan–Meier survival analysis (Figure 2 and Figure 3) stratified by EGFR/MGMT status revealed significant differences in survival outcomes among the four groups (log-rank test: *p* < 0.001). The EGFR neg/MGMT met group showed the best prognosis (median OS: 22.0 months, PFS: 10.5 months), whereas EGFR iper/MGMT unm had the worst (median OS: 10.0 months, PFS: 5.0 months). The survival advantage of MGMT methylation was evident in both non-amplified and amplified EGFR groups (HR: 0.45; 95% CI: 0.30–0.67, *p* < 0.001 for OS).

### 3.4. Recurrence Patterns and Correlation with Biomolecular Markers

Among the 81.2% of patients who experienced disease recurrence (Table 3), the distribution varied significantly by EGFR and MGMT status (Chi-square test: *p* = 0.002). In-field recurrence was most frequent in EGFR iper/MGMT unm patients (37.5%), while marginal and out-field recurrences were highest in EGFR iper/MGMT met patients (24.0%). Conversely, EGFR neg/MGMT met had the lowest rates of out-field recurrence (4.0%), suggesting a more localized recurrence pattern (Odds Ratio for out-field recurrence: 0.24; 95% CI: 0.12–0.48, *p* < 0.001).

### 3.5. Multivariate Cox Regression Analysis

To reduce the effect of potential confounders, we performed a multivariate Cox regression analysis including age, Karnofsky Performance Status (KPS), extent of resection (GTR, STR, biopsy), EGFR amplification, and MGMT promoter methylation status.

- MGMT methylation was significantly associated with improved OS (HR: 0.48; 95% CI: 0.33–0.69, *p* < 0.001) and PFS (HR: 0.54; 95% CI: 0.39–0.76, *p* = 0.001).
- EGFR amplification was independently associated with worse OS (HR: 1.57; 95% CI: 1.08–2.30, *p* = 0.02) and a higher likelihood of marginal recurrence (OR: 2.42; 95% CI: 1.22–4.81, *p* = 0.01).
- Gross total resection conferred a survival benefit (HR for OS: 0.67; 95% CI: 0.47–0.95, *p* = 0.03), whereas subtotal resection or biopsy was associated with earlier disease progression.
- The interaction term between EGFR amplification and MGMT methylation was not statistically significant (*p* = 0.09), suggesting that their effects on survival are largely independent.

### 3.6. Temporal Analysis of Recurrence

The median time to recurrence differed significantly between groups (log-rank *p* = 0.004):

- EGFR neg/MGMT met had the longest time to recurrence (median: 12.3 months).
- EGFR iper/MGMT unm had the shortest time to recurrence (median: 6.4 months).

Overall, these findings suggest that MGMT methylation is associated with improved survival but an increased risk of out-field recurrence, while EGFR amplification is linked to higher rates of marginal recurrence, potentially reflecting a more invasive tumor phenotype. These insights underscore the prognostic importance of EGFR and MGMT status in shaping glioblastoma treatment outcomes and recurrence dynamics.

### 3.7. Tumor Volume, Location and Biomolecular Markers

Baseline gross tumor volume (GTV) was available for all patients. The average GTV was 38.9 ± 14.2 cm^3^ across the cohort. No significant correlation was observed between initial tumor volume and MGMT promoter methylation (mean GTV: 37.8 ± 14.3 cm^3^ for methylated vs. 40.2 ± 13.9 cm^3^ for unmethylated tumors; *p* = 0.43), nor with EGFR amplification (mean GTV: 38.7 ± 14.1 cm^3^ for amplified vs. 39.5 ± 14.2 cm^3^ for non-amplified tumors; *p* = 0.61). Similarly, tumor location (frontal, temporal, parietal, deep nuclei) did not differ significantly among molecular subgroups (*p* > 0.05 for all comparisons). These findings suggest that baseline tumor volume and location is not associated with these biomolecular markers in our cohort.

### 3.8. Recurrence Therapy and Second Surgery

Of the 177 patients who experienced recurrence, 34 (15.6%) underwent a second surgical procedure. In terms of salvage treatment, 82 patients (37.6%) received second-line chemotherapy (mostly lomustine or regorafenib), 51 patients (23.4%) were treated with re-irradiation, and 9 (4.1%) received a second course of temozolomide. The remaining patients were managed with best supportive care or enrolled in experimental protocols. These post-progression therapies were heterogeneous and not evenly distributed across biomolecular subgroups.

## 4. Discussion

This retrospective cohort study suggests that the combined molecular profiling of MGMT promoter methylation and EGFR amplification may help to delineate prognostically distinct subgroups within the heterogeneous population of patients with IDH-wild-type glioblastoma (GBM). MGMT promoter methylation reflects epigenetic silencing of a DNA repair enzyme that removes alkyl adducts from the O6 position of guanine. This increases sensitivity to alkylating agents such as temozolomide and is currently the most validated biomarker used to guide treatment decisions in GBM, particularly in elderly or frail patients [5]. Our findings reaffirm the prognostic role of MGMT methylation and support its utility in identifying a biologically distinct subgroup with improved treatment response and more favorable prognosis. These subgroups appear to differ not only in survival outcomes, but also in patterns of radiological response and spatial recurrence. Such findings may offer preliminary insights into how molecular alterations could shape tumor behavior and inform therapeutic decision-making strategies [13].

One of the main observations of this analysis is that MGMT promoter methylation seems to be associated with a notable survival advantage (median OS 22.0 months vs. 10.0 months), in line with its previously reported predictive value for temozolomide (TMZ) sensitivity [22]. When stratified by both MGMT and EGFR status, the data suggest a more refined risk classification: the subgroup with MGMT methylation and no EGFR amplification showed the most favorable outcomes, including higher response rates and longer progression-free intervals. Conversely, the group with EGFR amplification and unmethylated MGMT exhibited the poorest prognosis, characterized by early progression and limited survival. These patterns point toward the potential added value of a dual-marker approach for prognostic stratification. EGFR amplification has been associated with increased tumor proliferation, invasiveness, and radioresistance [10]. Mechanistically, it activates key oncogenic pathways such as PI3K/AKT and Ras/MAPK, contributing to tumor progression. Although EGFR-targeted therapies have thus far shown limited efficacy in unselected GBM populations, emerging strategies such as antibody-drug conjugates and combination therapies may hold promise in molecularly defined subsets. Therefore, identifying EGFR amplification remains clinically relevant for future therapeutic stratification. The spatial analysis of recurrence may further support this perspective. Specifically, the combination of MGMT methylation and EGFR amplification was associated with a higher proportion of out-field recurrences (30.4%). This pattern may reflect a propensity for microscopic dissemination beyond conventional margins, particularly in tumors with longer survival allowing time for distant progression. These observations raise the possibility that patients with MGMT-methylated tumors might benefit from systemic treatment intensification or novel maintenance therapies aimed at reducing out-field failures. In contrast, the group characterized by EGFR amplification and unmethylated MGMT had the highest rate of marginal recurrences (24.2%), potentially indicating a locally infiltrative tumor phenotype [23,24]. This may suggest that standard clinical target volume (CTV) margins may be inadequate in this subgroup. A more tailored radiotherapy planning approach, possibly incorporating biological imaging or molecular profiling, could be warranted to better encompass the invasive front of the disease. These preliminary findings may warrant further exploration of biomarker-informed strategies for radiotherapy target definition, particularly regarding the adequacy of conventional treatment margins. From a clinical standpoint, such recurrence patterns could suggest the need for individualized adaptations of radiotherapy planning or systemic maintenance therapies, especially in molecularly high-risk subgroups. However, given the retrospective design, these observations should be interpreted with caution and require confirmation in prospective settings. Additionally, the divergent recurrence dynamics may be driven by underlying biological mechanisms, such as differences in tumor cell invasiveness, DNA repair efficiency, or microenvironmental interactions. This highlights the relevance of expanding molecular profiling beyond EGFR and MGMT—to include markers such as TERT, PTEN, or CDKN2A—which could refine risk stratification and potentially guide future therapeutic interventions [25]. The statistical analysis performed in this study also suggests that MGMT methylation and EGFR amplification act as independent prognostic variables. Although no significant interaction effect was observed (*p* = 0.09), their combined assessment may improve the overall prognostic resolution. MGMT methylation was associated with better OS and PFS (HR for OS: 0.48, *p* < 0.001), while EGFR amplification was related to poorer outcomes (HR for OS: 1.57, *p* = 0.02). Additionally, gross total resection (GTR) was associated with a survival benefit (HR: 0.67, *p* = 0.03), reinforcing the continued clinical importance of maximal surgical debulking in the management of GBM—even in the context of molecular classification. Notably, 15.6% of patients underwent a second surgical procedure at recurrence, which may have influenced post-progression survival and was not uniformly distributed among biomolecular subgroups. In summary, while our results are preliminary and constrained by the retrospective design, they support the potential clinical relevance of integrating MGMT and EGFR status into treatment planning and prognostication for IDH-wild-type GBM. These biomarkers may help identify subgroups who are at higher risk for specific patterns of failure, enabling more personalized approaches to radiotherapy and systemic therapy. Future prospective studies are essential to validate these findings and to establish risk-adapted, biomarker-driven therapeutic strategies in this patient population.

Further validation in prospective, multicenter studies is essential before such biomarker-driven approaches can be integrated into routine clinical practice.

## 5. Limitations

This study has several limitations. First, its retrospective design inherently introduces selection bias, as only patients with complete biomolecular and radiological data were included. The relatively low GTR rate (28.4%) may reflect the real-world difficulty of resection in deep-seated or eloquent-area tumors. In addition, some patients eligible for aggressive surgery may have been referred to clinical trials, potentially contributing to selection bias- Second, the single-center nature of the study may limit the generalizability of the findings, given potential institutional differences in surgical techniques, radiotherapy protocols, and supportive care. Third, while EGFR and MGMT were the focus of this analysis, other relevant molecular markers such as TERT promoter mutations, PTEN loss, and CDKN2A deletions were not assessed, potentially overlooking additional prognostic interactions. Fourth, the spatial classification of recurrences was based on co-registration with RT plans, which, despite meticulous methodology, carries inherent uncertainties due to anatomical changes over time and MRI variability. Finally, the relatively short median follow-up may have underestimated late recurrences or long-term survivors, and Quality-Adjusted Life Year (QALY) calculations were not feasible due to the retrospective nature of the study and lack of standardized quality-of-life assessments. Additionally, post-recurrence treatment strategies were heterogeneous and not controlled, which may have introduced confounding effects on survival outcomes across subgroup

## 6. Conclusions

In conclusion, this retrospective study suggests a potential prognostic value of EGFR amplification and MGMT promoter methylation in IDH-wild-type glioblastoma treated with standard chemoradiotherapy. MGMT methylation appears to be associated with improved survival, albeit with a higher frequency of out-field recurrences, while EGFR amplification may be linked to poorer outcomes and a more locally invasive recurrence pattern. These preliminary findings may support the rationale for further exploration of biomolecularly guided treatment adaptations, particularly in radiotherapy planning. Prospective and multicenter studies are warranted to validate these observations and better define their role in personalized management strategies for glioblastoma.

## Figures and Tables

**Table 1:** Patient demographics and clinical features, including age, sex, performance status, extent of resection, EGFR amplification, and MGMT methylation.

Variable	N of Patients (%)
Total patients	218
Age, mean (SD), years	61.8 (11.16)
Sex, male	141 (64.7%)
Sex, female	77 (35.3%)
Karnofsky Performance Status ≥80	193 (88.5%)
Karnofsky Performance Status <80	25 (11.5%)
Extent of resection: Gross Total	62 (28.4%)
Extent of resection: Subtotal	103 (47.2%)
Extent of resection: Biopsy	53 (24.3)
EGFR amplification (positive)	128 (58.7%)
EGFR amplification (negative)	90 (41.3%)
MGMT methylation (positive)	107 (49.1%)
MGMT methylation (negative)	111 (50.9%)

**Table 2:** Treatment response across EGFR/MGMT molecular subgroups (Chi-square test: *p* = 0.001).

Biomarker Group (EGFR/MGMT)	CR (%)	PR (%)	SD (%)	PD (%)	TOT
EGFR neg/MGMT met	16 (32.7%)	11 (22.4%)	10 (20.4%)	12 (24.5%)	49 (100%)
EGFR neg/MGMT unm	4 (10.3%)	11 (28.2%)	3 (7.7%)	21 (53.8%)	39 (100%)
EGFR iper/MGMT met	11 (23.9%)	9 (19.6%)	10 (21.7%)	26 (56.5%)	46 (100%)
EGFR iper/MGMT unm	8 (11.1%)	12 (16.7%)	9 (12.5%)	43 (59.7%)	72 (100%)

**Table 3:** Recurrence patterns across EGFR/MGMT molecular subgroups (Chi-square test: *p* = 0.002).

Biomarker Group (EGFR/MGMT)	In-Field Recurrence (%)	Marginal Recurrence (%)	Out-Field Recurrence (%)	TOT
EGFR neg/MGMT met	30 (81.1%)	1 (2.7%)	6 (16.2%)	37 (100%)
EGFR neg/MGMT unm	23 (82.1%)	2 (7.2%)	3 (10.7%)	28 (100%)
EGFR iper/MGMT met	26 (56.5%)	6 (13.1%)	14 (30.4%)	46 (100%)
EGFR iper/MGMT unm	47 (71.2%)	16 (24.2%)	3 (4.6%)	66 (100%)

## Data Availability

The data presented in this study are available on request from the corresponding author due to ethical reason.
